# Pyramidalization of a carbonyl C atom in (2*S*)-*N*-(seleno­acet­yl)proline methyl ester

**DOI:** 10.1107/S1600536813011112

**Published:** 2013-04-30

**Authors:** Ilia A. Guzei, Amit Choudhary, Ronald T. Raines

**Affiliations:** a Department of Chemistry, University of Wisconsin–Madison, 1101 University Ave, Madison, WI 53706-1322, USA; b Graduate Program in Biophysics, University of Wisconsin–Madison, 1525 Linden Drive, Madison, WI 53706-1534, USA; c Department of Biochemistry, University of Wisconsin–Madison, 433 Babcock Drive, Madison, WI 53706-1544, USA

## Abstract

The title compound, C_8_H_13_NO_2_Se, crystallizes as a non-merohedral twin with an approximate 9:1 component ratio with two symmetry-independent mol­ecules in the asymmetric unit. Our density-functional theory (DFT) computations indicate that the carb­oxy C atom is expected to be slightly pyramidal due to an *n*→ π* inter­action, wherein the lone pair (*n*) of the Se atom overlap with the anti­bonding orbital (π*) of the carbonyl group. Such pyramidalization is observed in one mol­ecule of the title compound but not the other.

## Related literature

For background to hybrid density functional theory (DFT) and natural bond orbital (NBO) analysis, see: Glendening *et al.* (2001 ▶); Weinhold (1998 ▶); Weinhold & Landis (2005 ▶). For literature related to the synthesis, see: Bhattacharyya & Woollins (2001 ▶) and for NBO studies of the title compound, see: Choudhary & Raines (2011*a* ▶); DeRider *et al.* (2002 ▶); Choudhary *et al.* (2009 ▶, 2010*a* ▶,*b* ▶); Jakobsche *et al.* (2010 ▶); Bartlett *et al.* (2010 ▶); Choudhary & Raines (2011*b* ▶). For geometrical checks with *ConQuest* and *Mercury*, see: Bruno *et al.* (2002 ▶). For *Gaussian 03* software, see: Frisch (2004 ▶). For puckering parameters, see: Cremer & Pople (1975 ▶).
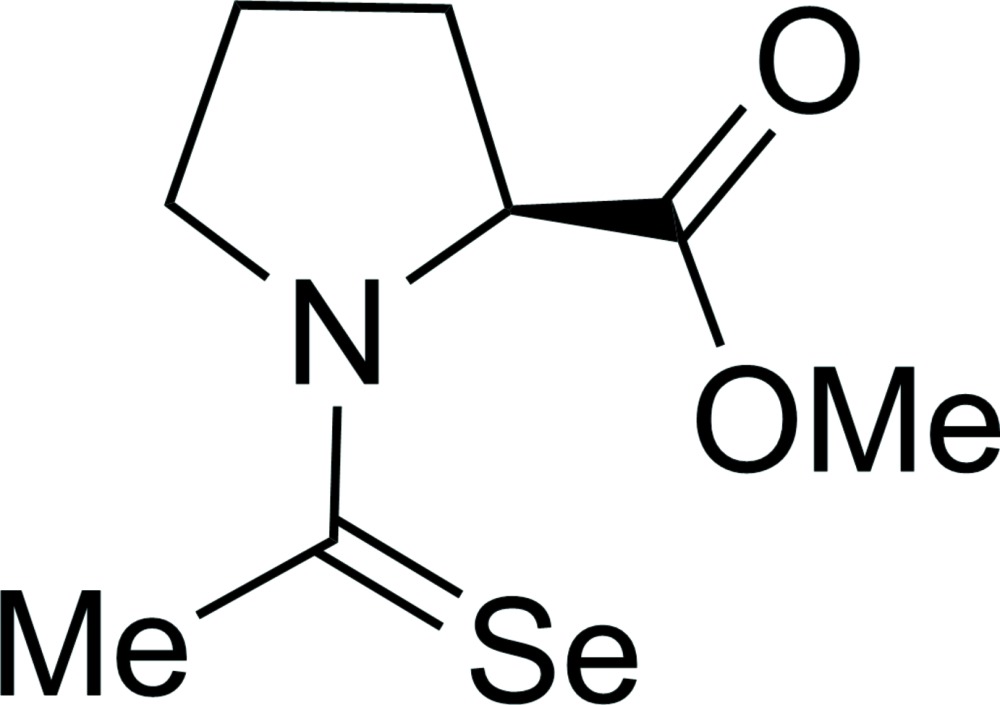


## Experimental

### 

#### Crystal data

C_8_H_13_NO_2_Se
*M* *_r_* = 234.15
Triclinic, 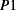

*a* = 7.050 (3) Å
*b* = 7.442 (3) Å
*c* = 10.334 (4) Å
α = 85.166 (6)°
β = 86.220 (6)°
γ = 64.682 (4)°
*V* = 488.1 (3) Å^3^
*Z* = 2
Mo *K*α radiation
μ = 3.81 mm^−1^
*T* = 105 K
0.47 × 0.37 × 0.35 mm

#### Data collection

Bruker SMART APEX2 area detector diffractometer
Absorption correction: multi-scan (*TWINABS*; Bruker, 2007 ▶) *T*_min_ = 0.268, *T*_max_ = 0.349
3012 measured reflections
3012 independent reflections
2938 reflections with *I* > 2σ(*I*)

#### Refinement

*R*[*F* ^2^ > 2σ(*F* ^2^)] = 0.051
*wR*(*F* ^2^) = 0.135
*S* = 1.12
3012 reflections
224 parameters
3 restraints
H-atom parameters constrained
Δρ_max_ = 1.46 e Å^−3^
Δρ_min_ = −0.63 e Å^−3^
Absolute structure: Classical Flack method preferred over Parsons because s.u. lower.
Flack parameter: 0.01 (3)

### 

Data collection: *APEX2* (Bruker, 2012 ▶); cell refinement: *SAINT-Plus* (Bruker, 2007 ▶); data reduction: *SAINT-Plus*; program(s) used to solve structure: *SHELXTL* (Sheldrick, 2008 ▶); program(s) used to refine structure: *SHELXTL*; molecular graphics: *OLEX2* (Dolomanov *et al.*, 2009 ▶) and *NBOView* (Wendt & Weinhold, 2001 ▶); software used to prepare material for publication: *OLEX2*, *GX* and FCF_filter (Guzei, 2012 ▶).

## Supplementary Material

Crystal structure: contains datablock(s) global, I. DOI: 10.1107/S1600536813011112/kj2221sup1.cif

Structure factors: contains datablock(s) I. DOI: 10.1107/S1600536813011112/kj2221Isup2.hkl

Supplementary material file. DOI: 10.1107/S1600536813011112/kj2221Isup3.cml

Additional supplementary materials:  crystallographic information; 3D view; checkCIF report

## Figures and Tables

**Fig. 1.**
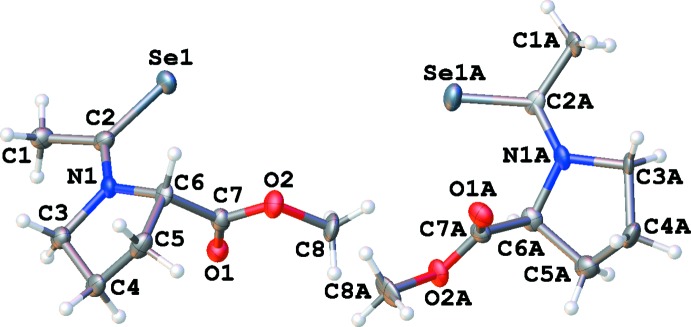
Molecular structure of (I). The thermal ellipsoids are shown at 50% probability level.

**Fig. 2.**
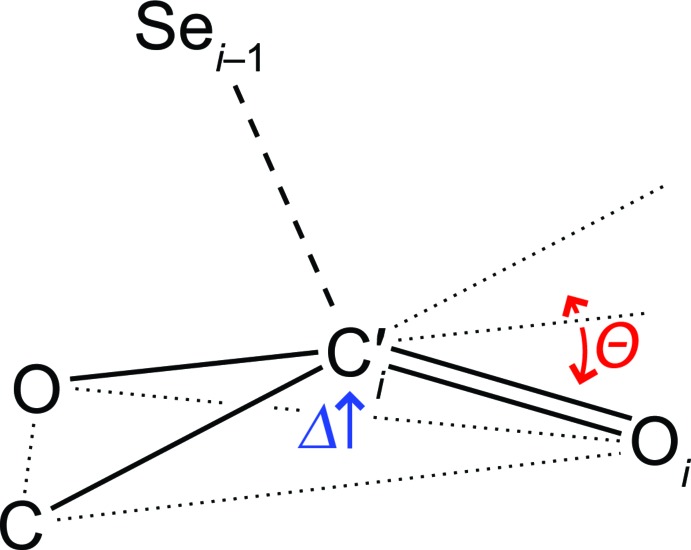
Pyramidalization parameters Δ and Θ of an n→π* interaction in (I).

**Fig. 3.**
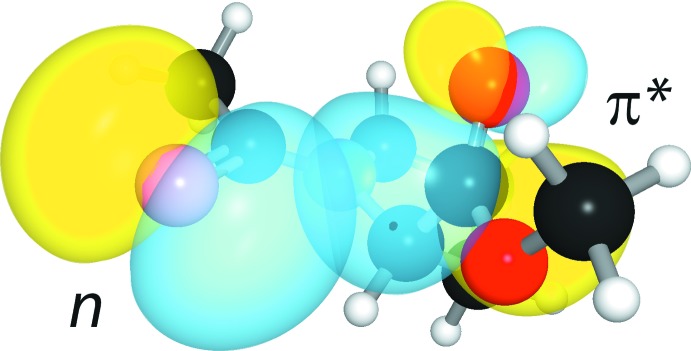
An NBO depiction of the n→π* orbital overlap in (I) generated with NBOView (Wendt & Weinhold, 2001)

## supplementary crystallographic information

### Comment

We have previously reported on extensive studies of geometrical and conformational attributes of several amide bond isosteres (Choudhary & Raines, 2011*a*). In contrast, selenoamides have not received much attention. Herein we report the crystal structure of the title compound *N*-selenoacetyl–(2*S*)-proline methyl ester (Scheme 1, (I)) and the results of a hybrid density functional theory (DFT) and Natural Bond Orbital (NBO) analysis (Glendening *et al.*, 2001, Weinhold, 1998, Weinhold & Landis, 2005) of its geometrical features.

Compound (I), Figure 1, crystallizes as a non-merohedral twin with the minor component contribution of 10 (2)%. The two components are related by 179° degree rotation about the [110] vector. The asymmetric unit in the relatively rare space group P1 contains two symmetry-independent molecules with the same handedness. The absolute structures of both components have been unequivocally established by anomalous dispersion effects: the Flack *x* parameters for the two components have been refined independently to 0.04 (2) and 0.02 (2). The two molecules have essentially identical geometries and their non-H atoms can be superimposed with a RMS of 0.042 Å. All geometrical parameters in the molecules are typical within experimental error (Bruno *et al.*, 2002). The conformations of the five-membered rings in (I) are characterized by the puckering coordinates (Cremer & Pople, 1975) q_2_ and φ_2_ which measured 0.376 (14) Å and 89.8 (19)° for the Se1 molecule and 0.370 (15) Å and 85 (2)° for the Se1a molecule. Whereas the extent of puckering of the rings is the same, the ring in the Se1 molecule is in twisted conformation ^3^T_4_ whereas the ring in the other molecule is in the ^3^T_4_ conformation with a small ^3^E envelope character. The envelope character is probably statistically significant.

The key feature of (I) is pyramidalization of atom C7 described with parameters Δ and Θ defined in Figure 2. These parameters are 0.016 (12) Å and 0.06 (5)° for the Se1 molecule and 0.040 (13) Å and 1.5 (5)° for the Se1a molecule. In the Se1 molecule the pyramidalization is not observed whereas in the second molecule the slight pyramidalization is statistically significant. For comparison, in the sulfur analog of (I) the relevant pyramidalization parameters Δ and Θ are 0.0293 (13) Å and 1.10 (5)°, also small and statistically significant.

We conducted DFT and NBO analyses of four low energy conformations of (I) (DeRider *et al.*, 2002, Choudhary *et al.*, 2009, Choudhary *et al.*, 2010*b*, Choudhary *et al.*, 2010*a*, Jakobsche *et al.*, 2010) at the B3LYP/6–311+G(2 d,p) level of theory using Gaussian 03 (Frisch *et al.*, 2004) and comment here on the most stable conformer. We have previously reported an interaction in proteins, termed the *n*→π* interaction, wherein the lone pairs (*n*) of an oxygen (O_i-1_) of a carbonyl group overlap with the antibonding orbital (π*) of C_i_=O_i_ of an adjacent carbonyl group. The similar overlap in (I) between the lone pairs (*n*) of the selenium and the antibonding orbital (π*) of the carbonyl group is shown in Figure 3. (Bartlett *et al.*, 2010, Choudhary & Raines, 2011*b*). This interaction resembles the Bürgi–Dunitz trajectory for nucleophilic additions to the carbonyl group and induces pyramidalization of the acceptor carbonyl group (Choudhary *et al.*, 2009). The second-order perturbation theory as implemented in NBO 5.0 suggests *n*→π* interaction mediated stabilization of the *trans* conformation by 0.84 kcal/mol. The findings of our crystallographic studies partially support our theoretical findings: molecule Se1A shows pyramidalization whereas molecule Se1 does not. We attribute these results to the twinned nature of the crystals that lead to relatively high e.s.d.'s on geometrical parameters, but it was not possible to isolate a better crystal.

### Experimental

Compound (I) was synthesized following from its oxygen congener by using (PhPSe_2_)_2_ (Woolins' reagent) following a procedure reported previously (Bhattacharyya & Woollins, 2001). A small amount of (I) was dissolved in hexanes with a minimal amount of ethyl acetate. Slow evaporation of the solution afforded X-ray quality crystals of (I) after ~4 days.

### Refinement

All H-atoms were placed in idealized locations and refined as riding with appropriate thermal displacement coefficients *U*_iso_(H) = 1.2 or 1.5 times *U*_eq_(bearing atom).

### Crystal data

**Table:**

C_8_H_13_NO_2_Se	*Z* = 2
*M**_r_* = 234.15	*F*(000) = 236
Triclinic, *P*1	*D*_x_ = 1.593 Mg m^−^^3^
*a* = 7.050 (3) Å	Mo *K*α radiation, λ = 0.71073 Å
*b* = 7.442 (3) Å	Cell parameters from 746 reflections
*c* = 10.334 (4) Å	θ = 3.0–29.0°
α = 85.166 (6)°	µ = 3.81 mm^−^^1^
β = 86.220 (6)°	*T* = 105 K
γ = 64.682 (4)°	Block, colourless
*V* = 488.1 (3) Å^3^	0.47 × 0.37 × 0.35 mm

### Data collection

**Table:**

Bruker SMART APEX2 area detector diffractometer	3012 measured reflections
Radiation source: microfocus sealed X-ray tube, Incoatec Iµs	3012 independent reflections
Mirror optics monochromator	2938 reflections with *I* > 2σ(*I*)
Detector resolution: 7.9 pixels mm^-1^	θ_max_ = 25.1°, θ_min_ = 2.0°
0.5° ω and 0.5° φ scans	*h* = −8→8
Absorption correction: multi-scan (TWINABS; Bruker, 2007)	*k* = −8→8
*T*_min_ = 0.268, *T*_max_ = 0.349	*l* = −12→12

### Refinement

**Table:**

Refinement on *F*^2^	Hydrogen site location: inferred from neighbouring sites
Least-squares matrix: full	H-atom parameters constrained
*R*[*F*^2^ > 2σ(*F*^2^)] = 0.051	*w* = 1/[σ^2^(*F*_o_^2^) + (0.0947*P*)^2^ + 1.2373*P*] where *P* = (*F*_o_^2^ + 2*F*_c_^2^)/3
*wR*(*F*^2^) = 0.135	(Δ/σ)_max_ < 0.001
*S* = 1.12	Δρ_max_ = 1.46 e Å^−^^3^
3012 reflections	Δρ_min_ = −0.63 e Å^−^^3^
224 parameters	Absolute structure: Classical Flack method preferred over Parsons because s.u. lower.
3 restraints	Flack parameter: 0.01 (3)

### Special details

**Table:**

Geometry. All e.s.d.'s (except the e.s.d. in the dihedral angle between two l.s. planes) are estimated using the full covariance matrix. The cell e.s.d.'s are taken into account individually in the estimation of e.s.d.'s in distances, angles and torsion angles; correlations between e.s.d.'s in cell parameters are only used when they are defined by crystal symmetry. An approximate (isotropic) treatment of cell e.s.d.'s is used for estimating e.s.d.'s involving l.s. planes.
Refinement. Refined as a 4-component twin.

### Fractional atomic coordinates and isotropic or equivalent isotropic displacement parameters (Å^2^)

**Table:**

	*x*	*y*	*z*	*U*_iso_*/*U*_eq_	
Se1	1.02636 (11)	0.03198 (10)	0.80590 (9)	0.0268 (3)	
O1	0.6582 (13)	0.5319 (12)	0.9097 (8)	0.0258 (17)	
O2	0.6687 (15)	0.5834 (13)	0.6919 (8)	0.0267 (19)	
N1	0.6036 (14)	0.1857 (14)	0.8793 (9)	0.0209 (18)	
C1	0.807 (2)	−0.108 (2)	1.0113 (14)	0.024 (3)	
H1A	0.7617	−0.0430	1.0937	0.036*	
H1B	0.9523	−0.2093	1.0174	0.036*	
H1C	0.7156	−0.1708	0.9932	0.036*	
C2	0.7948 (17)	0.0453 (16)	0.9034 (12)	0.023 (2)	
C3	0.4089 (18)	0.2096 (19)	0.9573 (12)	0.024 (3)	
H3A	0.4309	0.2039	1.0515	0.029*	
H3B	0.3636	0.1052	0.9401	0.029*	
C4	0.2498 (18)	0.4141 (18)	0.9102 (13)	0.027 (3)	
H4A	0.1058	0.4219	0.9172	0.032*	
H4B	0.2550	0.5190	0.9608	0.032*	
C5	0.3161 (18)	0.436 (2)	0.7695 (13)	0.028 (3)	
H5A	0.2615	0.5776	0.7380	0.034*	
H5B	0.2662	0.3647	0.7132	0.034*	
C6	0.5599 (17)	0.3388 (16)	0.7724 (10)	0.020 (2)	
H6	0.6241	0.2781	0.6885	0.024*	
C7	0.6369 (17)	0.4933 (16)	0.8033 (11)	0.022 (2)	
C8	0.734 (3)	0.740 (2)	0.7072 (15)	0.031 (3)	
H8A	0.6159	0.8558	0.7411	0.046*	
H8B	0.7828	0.7783	0.6228	0.046*	
H8C	0.8493	0.6918	0.7681	0.046*	
Se1A	1.07753 (15)	0.71492 (14)	0.39148 (11)	0.0314 (4)	
O1A	0.5592 (13)	1.1157 (14)	0.2797 (8)	0.0279 (18)	
O2A	0.5329 (13)	1.1213 (14)	0.4976 (9)	0.0264 (19)	
N1A	0.9670 (14)	1.1015 (14)	0.2881 (9)	0.0212 (19)	
C1A	1.259 (2)	0.861 (2)	0.1724 (16)	0.026 (3)	
H1AA	1.1935	0.9144	0.0886	0.038*	
H1AB	1.3372	0.7162	0.1715	0.038*	
H1AC	1.3562	0.9199	0.1874	0.038*	
C2A	1.0943 (16)	0.9115 (16)	0.2781 (11)	0.020 (2)	
C3A	0.9718 (18)	1.2692 (17)	0.2000 (12)	0.025 (2)	
H3AA	0.9716	1.2428	0.1077	0.030*	
H3AB	1.0970	1.2921	0.2141	0.030*	
C4A	0.771 (2)	1.446 (2)	0.2387 (14)	0.031 (3)	
H4AA	0.7858	1.5722	0.2220	0.037*	
H4AB	0.6505	1.4540	0.1904	0.037*	
C5A	0.7423 (19)	1.4030 (19)	0.3828 (13)	0.031 (3)	
H5AA	0.5938	1.4753	0.4120	0.037*	
H5AB	0.8312	1.4416	0.4341	0.037*	
C6A	0.8117 (17)	1.1760 (18)	0.3963 (11)	0.022 (2)	
H6A	0.8771	1.1185	0.4819	0.027*	
C7A	0.6233 (17)	1.1279 (17)	0.3787 (11)	0.023 (2)	
C8A	0.347 (2)	1.085 (3)	0.4944 (16)	0.040 (4)	
H8AA	0.2399	1.1934	0.4433	0.060*	
H8AB	0.2912	1.0772	0.5832	0.060*	
H8AC	0.3832	0.9585	0.4546	0.060*	

### Atomic displacement parameters (Å^2^)

**Table:**

	*U*^11^	*U*^22^	*U*^33^	*U*^12^	*U*^13^	*U*^23^
Se1	0.0198 (6)	0.0286 (7)	0.0363 (8)	−0.0150 (6)	0.0045 (5)	−0.0029 (5)
O1	0.028 (4)	0.027 (4)	0.026 (5)	−0.016 (4)	0.000 (3)	−0.004 (3)
O2	0.030 (5)	0.030 (5)	0.029 (5)	−0.021 (4)	−0.007 (4)	0.000 (3)
N1	0.020 (5)	0.021 (5)	0.026 (5)	−0.014 (4)	0.002 (3)	−0.001 (3)
C1	0.019 (6)	0.023 (7)	0.027 (7)	−0.007 (6)	−0.004 (5)	−0.001 (5)
C2	0.016 (5)	0.019 (5)	0.037 (7)	−0.008 (5)	0.000 (4)	−0.006 (4)
C3	0.020 (6)	0.022 (6)	0.035 (6)	−0.012 (5)	0.001 (5)	−0.004 (5)
C4	0.015 (5)	0.021 (6)	0.047 (7)	−0.011 (5)	0.001 (5)	−0.004 (5)
C5	0.021 (6)	0.020 (6)	0.045 (7)	−0.009 (5)	−0.010 (5)	0.002 (5)
C6	0.021 (5)	0.022 (5)	0.024 (5)	−0.015 (5)	−0.006 (4)	−0.002 (4)
C7	0.018 (5)	0.023 (6)	0.029 (6)	−0.011 (5)	−0.003 (4)	0.001 (4)
C8	0.039 (8)	0.025 (7)	0.039 (8)	−0.025 (6)	0.000 (6)	0.001 (5)
Se1A	0.0285 (7)	0.0247 (7)	0.0453 (9)	−0.0164 (6)	−0.0005 (6)	0.0041 (6)
O1A	0.019 (4)	0.039 (5)	0.030 (5)	−0.016 (4)	−0.001 (3)	−0.005 (4)
O2A	0.019 (4)	0.035 (5)	0.033 (5)	−0.019 (4)	−0.005 (3)	0.002 (3)
N1A	0.018 (5)	0.029 (5)	0.023 (5)	−0.018 (4)	−0.002 (3)	0.002 (4)
C1A	0.018 (6)	0.027 (7)	0.039 (8)	−0.018 (6)	0.003 (5)	0.000 (6)
C2A	0.015 (5)	0.022 (6)	0.028 (6)	−0.011 (5)	−0.012 (4)	0.000 (4)
C3A	0.022 (6)	0.019 (5)	0.039 (7)	−0.015 (5)	0.002 (5)	0.007 (5)
C4A	0.026 (7)	0.016 (7)	0.051 (8)	−0.011 (6)	−0.002 (6)	0.003 (6)
C5A	0.022 (6)	0.030 (7)	0.043 (8)	−0.013 (6)	0.004 (5)	−0.009 (6)
C6A	0.017 (5)	0.029 (7)	0.024 (6)	−0.012 (5)	−0.006 (4)	0.000 (5)
C7A	0.020 (6)	0.027 (6)	0.026 (6)	−0.014 (5)	0.004 (4)	−0.006 (4)
C8A	0.027 (7)	0.068 (12)	0.043 (8)	−0.038 (8)	0.010 (6)	−0.013 (8)

### Geometric parameters (Å, º)

**Table:**

Se1—C2	1.831 (11)	Se1A—C2A	1.835 (11)
O1—C7	1.194 (14)	O1A—C7A	1.170 (14)
O2—C7	1.337 (14)	O2A—C7A	1.354 (15)
O2—C8	1.450 (16)	O2A—C8A	1.454 (16)
N1—C2	1.329 (15)	N1A—C2A	1.319 (15)
N1—C3	1.495 (14)	N1A—C3A	1.493 (14)
N1—C6	1.465 (14)	N1A—C6A	1.477 (15)
C1—H1A	0.9800	C1A—H1AA	0.9800
C1—H1B	0.9800	C1A—H1AB	0.9800
C1—H1C	0.9800	C1A—H1AC	0.9800
C1—C2	1.504 (19)	C1A—C2A	1.490 (18)
C3—H3A	0.9900	C3A—H3AA	0.9900
C3—H3B	0.9900	C3A—H3AB	0.9900
C3—C4	1.516 (18)	C3A—C4A	1.522 (18)
C4—H4A	0.9900	C4A—H4AA	0.9900
C4—H4B	0.9900	C4A—H4AB	0.9900
C4—C5	1.514 (19)	C4A—C5A	1.51 (2)
C5—H5A	0.9900	C5A—H5AA	0.9900
C5—H5B	0.9900	C5A—H5AB	0.9900
C5—C6	1.555 (16)	C5A—C6A	1.541 (17)
C6—H6	1.0000	C6A—H6A	1.0000
C6—C7	1.529 (15)	C6A—C7A	1.541 (15)
C8—H8A	0.9800	C8A—H8AA	0.9800
C8—H8B	0.9800	C8A—H8AB	0.9800
C8—H8C	0.9800	C8A—H8AC	0.9800
			
C7—O2—C8	114.7 (10)	C7A—O2A—C8A	113.3 (10)
C2—N1—C3	124.9 (10)	C2A—N1A—C3A	125.3 (9)
C2—N1—C6	123.3 (9)	C2A—N1A—C6A	123.3 (10)
C6—N1—C3	111.8 (9)	C6A—N1A—C3A	111.1 (9)
H1A—C1—H1B	109.5	H1AA—C1A—H1AB	109.5
H1A—C1—H1C	109.5	H1AA—C1A—H1AC	109.5
H1B—C1—H1C	109.5	H1AB—C1A—H1AC	109.5
C2—C1—H1A	109.5	C2A—C1A—H1AA	109.5
C2—C1—H1B	109.5	C2A—C1A—H1AB	109.5
C2—C1—H1C	109.5	C2A—C1A—H1AC	109.5
N1—C2—Se1	122.0 (9)	N1A—C2A—Se1A	122.3 (9)
N1—C2—C1	115.8 (10)	N1A—C2A—C1A	117.2 (11)
C1—C2—Se1	122.2 (8)	C1A—C2A—Se1A	120.4 (9)
N1—C3—H3A	111.1	N1A—C3A—H3AA	111.1
N1—C3—H3B	111.1	N1A—C3A—H3AB	111.1
N1—C3—C4	103.2 (10)	N1A—C3A—C4A	103.2 (9)
H3A—C3—H3B	109.1	H3AA—C3A—H3AB	109.1
C4—C3—H3A	111.1	C4A—C3A—H3AA	111.1
C4—C3—H3B	111.1	C4A—C3A—H3AB	111.1
C3—C4—H4A	111.0	C3A—C4A—H4AA	111.0
C3—C4—H4B	111.0	C3A—C4A—H4AB	111.0
H4A—C4—H4B	109.0	H4AA—C4A—H4AB	109.0
C5—C4—C3	104.0 (10)	C5A—C4A—C3A	103.9 (11)
C5—C4—H4A	111.0	C5A—C4A—H4AA	111.0
C5—C4—H4B	111.0	C5A—C4A—H4AB	111.0
C4—C5—H5A	111.1	C4A—C5A—H5AA	111.0
C4—C5—H5B	111.1	C4A—C5A—H5AB	111.0
C4—C5—C6	103.3 (10)	C4A—C5A—C6A	103.8 (9)
H5A—C5—H5B	109.1	H5AA—C5A—H5AB	109.0
C6—C5—H5A	111.1	C6A—C5A—H5AA	111.0
C6—C5—H5B	111.1	C6A—C5A—H5AB	111.0
N1—C6—C5	102.9 (9)	N1A—C6A—C5A	103.7 (9)
N1—C6—H6	111.1	N1A—C6A—H6A	110.9
N1—C6—C7	110.4 (8)	N1A—C6A—C7A	110.0 (9)
C5—C6—H6	111.1	C5A—C6A—H6A	110.9
C7—C6—C5	110.0 (9)	C5A—C6A—C7A	110.1 (9)
C7—C6—H6	111.1	C7A—C6A—H6A	110.9
O1—C7—O2	125.6 (10)	O1A—C7A—O2A	125.9 (11)
O1—C7—C6	125.5 (10)	O1A—C7A—C6A	126.3 (10)
O2—C7—C6	108.8 (9)	O2A—C7A—C6A	107.6 (9)
O2—C8—H8A	109.5	O2A—C8A—H8AA	109.5
O2—C8—H8B	109.5	O2A—C8A—H8AB	109.5
O2—C8—H8C	109.5	O2A—C8A—H8AC	109.5
H8A—C8—H8B	109.5	H8AA—C8A—H8AB	109.5
H8A—C8—H8C	109.5	H8AA—C8A—H8AC	109.5
H8B—C8—H8C	109.5	H8AB—C8A—H8AC	109.5
			
N1—C3—C4—C5	31.5 (11)	N1A—C3A—C4A—C5A	32.7 (12)
N1—C6—C7—O1	−25.3 (15)	N1A—C6A—C7A—O1A	−30.5 (16)
N1—C6—C7—O2	157.1 (9)	N1A—C6A—C7A—O2A	155.3 (9)
C2—N1—C3—C4	167.0 (10)	C2A—N1A—C3A—C4A	169.8 (10)
C2—N1—C6—C5	169.2 (10)	C2A—N1A—C6A—C5A	166.9 (9)
C2—N1—C6—C7	−73.5 (12)	C2A—N1A—C6A—C7A	−75.3 (12)
C3—N1—C2—Se1	−177.9 (8)	C3A—N1A—C2A—Se1A	179.8 (8)
C3—N1—C2—C1	5.1 (16)	C3A—N1A—C2A—C1A	1.6 (15)
C3—N1—C6—C5	−11.6 (11)	C3A—N1A—C6A—C5A	−8.4 (11)
C3—N1—C6—C7	105.7 (10)	C3A—N1A—C6A—C7A	109.4 (10)
C3—C4—C5—C6	−39.0 (11)	C3A—C4A—C5A—C6A	−38.3 (12)
C4—C5—C6—N1	30.9 (11)	C4A—C5A—C6A—N1A	28.6 (11)
C4—C5—C6—C7	−86.8 (11)	C4A—C5A—C6A—C7A	−89.0 (11)
C5—C6—C7—O1	87.6 (14)	C5A—C6A—C7A—O1A	83.2 (15)
C5—C6—C7—O2	−90.1 (11)	C5A—C6A—C7A—O2A	−91.0 (11)
C6—N1—C2—Se1	1.2 (14)	C6A—N1A—C2A—Se1A	5.2 (14)
C6—N1—C2—C1	−175.8 (11)	C6A—N1A—C2A—C1A	−173.0 (11)
C6—N1—C3—C4	−12.1 (12)	C6A—N1A—C3A—C4A	−15.0 (12)
C8—O2—C7—O1	0.0 (18)	C8A—O2A—C7A—O1A	3.5 (18)
C8—O2—C7—C6	177.7 (10)	C8A—O2A—C7A—C6A	177.7 (11)
